# Bioinformatics Analysis Explores Potential Hub Genes in Nonalcoholic Fatty Liver Disease

**DOI:** 10.3389/fgene.2021.772487

**Published:** 2021-10-29

**Authors:** Chutian Wu, Yun Zhou, Min Wang, Guolin Dai, Xiongxiu Liu, Leizhen Lai, Shaohui Tang

**Affiliations:** ^1^ Department of Gastroenterology, The First Affiliated Hospital, Jinan University, Guangzhou, China; ^2^ Department of Gastroenterology, The First Affiliated Hospital, Gannan Medical University, Ganzhou, China

**Keywords:** nonalcoholic fatty liver disease, nonalcoholic steatohepatitis, differentially expressed genes, hepatocellular carcinoma, bioinformatics analysis

## Abstract

**Background:** Nonalcoholic fatty liver disease (NAFLD) is now recognized as the most prevalent chronic liver disease worldwide. However, the dysregulated gene expression for NAFLD is still poorly understood.

**Material and methods:** We analyzed two public datasets (GSE48452 and GSE89632) to identify differentially expressed genes (DEGs) in NAFLD. Then, we performed a series of bioinformatics analyses to explore potential hub genes in NAFLD.

**Results:** This study included 26 simple steatosis (SS), 34 nonalcoholic steatohepatitis (NASH), and 13 healthy controls (HC). We observed 6 up- and 19 down-regulated genes in SS, and 13 up- and 19 down-regulated genes in NASH compared with HC. Meanwhile, the overlapping pathways between SS and NASH were PI3K-Akt signaling pathway and pathways in cancer. Then, we screened out 10 hub genes by weighted Gene Co-Expression Network Analysis (WGCNA) and protein-protein interaction (PPI) networks. Eventually, we found that *CYP7A1*/*GINS2*/*PDLIM3* were associated with the prognosis of hepatocellular carcinoma (HCC) in the TCGA database.

**Conclusion:** Although further validation is still needed, we provide useful and novel information to explore the potential candidate genes for NAFLD prognosis and therapeutic options.

## Introduction

Nonalcoholic fatty liver disease (NAFLD) is now recognized as the most prevalent chronic liver disease worldwide, with a prevalence ranging from 13% in Africa to 42% in southeast Asia, and it may become the major cause of end-stage liver diseases by 2025 (Zarrinpar et al., 2016; Younossi, 2019; Huang et al., 2021). NAFLD represents a spectrum of disease severity, ranging from simple steatosis (SS) termed as nonalcoholic fatty liver (NAFL) to nonalcoholic steatohepatitis (NASH), cirrhosis, and hepatocellular carcinoma (HCC) (Natarajan et al., 2014). It has been well-recognized that obesity, insulin resistance, and type 2 diabetes mellitus are the strongest risk factors for NAFLD (Chen and Tian, 2020). The cause of NAFLD is multifactorial, including genetic and environmental factors (Chen and Tian, 2020). However, possible effects and underlying mechanisms for NAFLD are still not understood. Meanwhile, NAFLD-related HCC usually lacks symptoms and tends to be diagnosed at a later stage and is related to poorer survival than viral hepatitis-related HCC (Younossi et al., 2015; Huang et al., 2021). In addition, NAFLD-related HCC is now proliferating and will increase in parallel with the obesity epidemic (Desai et al., 2019). Therefore, it is essential to investigate in detail the mechanism in the pathogenesis of NAFLD to find new potential targets for prognosis and therapy, especially in obese population.

Many genome-wide association studies have indicated that *PNPLA3*, *HNF1A*, *NCAN*, *GCKR*, *MBOTAT*, *FADS1*, *PPAR*, *TNF*, and *TM6SF2* are important genetic and epigenetic modifiers played important roles in the pathogenesis and progression of NAFLD (Choudhary and Duseja, 2021). Meanwhile, some bioinformatics researches offer new ideas for exploring potential targets of NAFLD. Zeng et al. (2020) found that *AKR1B10* and *SPP1* were related to immune cell infiltrations and associated with NAFLD progression. Liu et al. (2020) reported that *TOP2A*, *NHP2L1*, *PCNA*, *CHEK1*, *ACACA*, *CCS*, *ACACB* had a significant impact on NAFLD progression and were associated with HCC progression. What’s more, Wang et al. (2016) indicated that *Lp1*, *Ces2*, *Fasn*, *Hmgcs1*, *Sc4mol*, *Fads1*, and *Mup1* were associated with lipid metabolism, and *Cbr3*, *Trib3*, *Nfe212* were related to oxidative stress in NAFLD mouse model. Obviously, there is significant heterogeneity between studies in both animal and human experiments. Although many studies have been devoted to exploring the pathogenesis and progression of NAFLD, there are still no effective drugs for the treatment of NAFLD except for lifestyle changes (Leoni et al., 2018). Thus, combination bioinformatics analysis with public microarray data will contribute to explore novel pathways and genes regulating NAFLD.

Therefore, we analyzed two public datasets to identify differentially expressed genes (DEGs) among healthy controls (HC), SS, and NASH. Then, Weighted Gene Co-Expression Network Analysis (WGCNA) and protein-protein interaction (PPI) networks were performed to explore the impact of DEGs on NAFLD. This study aimed to screen potential genes for NAFLD development.

## Material and Methods

### Data Retrieving and Processing

The gene expression profiles of GSE48452 (Ahrens et al., 2013) and GSE89632 (Arendt et al., 2015) were downloaded from Gene Expression Omnibus (GEO, http://www.ncbi.nlm.nih.gov/geo). To prevent the effects of overweight in the evaluation, healthy obesity with body mass index (BMI) over 24 kg/m^2^ were excluded from the HC group. Besides, due to NAFLD commonly happened to the obese population, NALFD patients with BMI less than 24 kg/m^2^ were also excluded from the experimental group. What is more, individuals with bariatric surgery or severely missing data at baseline were also ruled out. Finally, 9 SS samples, 17 NASH samples, and 5 HC samples in the GSE48452, and 17 SS samples, 17 NASH samples, and 8 HC samples in the GSE89632 were included in this study (Table 1). HCC data were obtained from The Cancer Genome Atlas (TCGA) database, including 374 HCC samples and 50 normal samples.

**TABLE 1:** The data are shown as median (interquartile range, IQR). HC, healthy control; SS, simple steatosis; NASH, nonalcoholic steatohepatitis; BMI, body mass index; NAS, NAFLD activity score.

**Dataset**	**HC**	**SS**	**NASH**
GSE48452 (n)	5	9	17
Gender (male: female)	0:5	2:7	4:13
Age (years)	45.0 (35.0–62.0)	37.0 (32.0–46.5)	47 (36–50.5)
BMI (kg/m^2^)	21.0 (18.8–23.5)	51.9 (45.7–55.7)	47.8 (33.4–55.7)
Steatosis (%)	0 (0–2.0)	30.0 (15.0–70.0)	75 (70.0–85.0)
NAS	0.5 (0–1.0)	1.0 (1.0–3.0)	5.0 (5.0–5.5)
GSE89632 (n)	8	17	17
Gender (male: female)	4:4	12:5	9:8
Age (years)	42.5 (26.5–54.3)	45.0 (35–51.5)	44.0 (35.5–52.5)
BMI (kg/m^2^)	21.2 (19.9–23.1)	28.9 (27.5–31.3)	32.0 (29.65–33.6)
Steatosis (%)	0 (0–0.8)	40.0 (15.0–55.0)	40.0 (17.5–70.0)
NAS	0 (0–0)	2.0 (1.0–2.0)	5.0 (4.0–6.0)

The data are shown as mean and median (interquartile range, IQR). HC, healthy control; SS, simple steatosis; NASH, nonalcoholic steatohepatitis; BMI, body mass index; NAS, NAFLD activity score.

**FIGURE 1:**
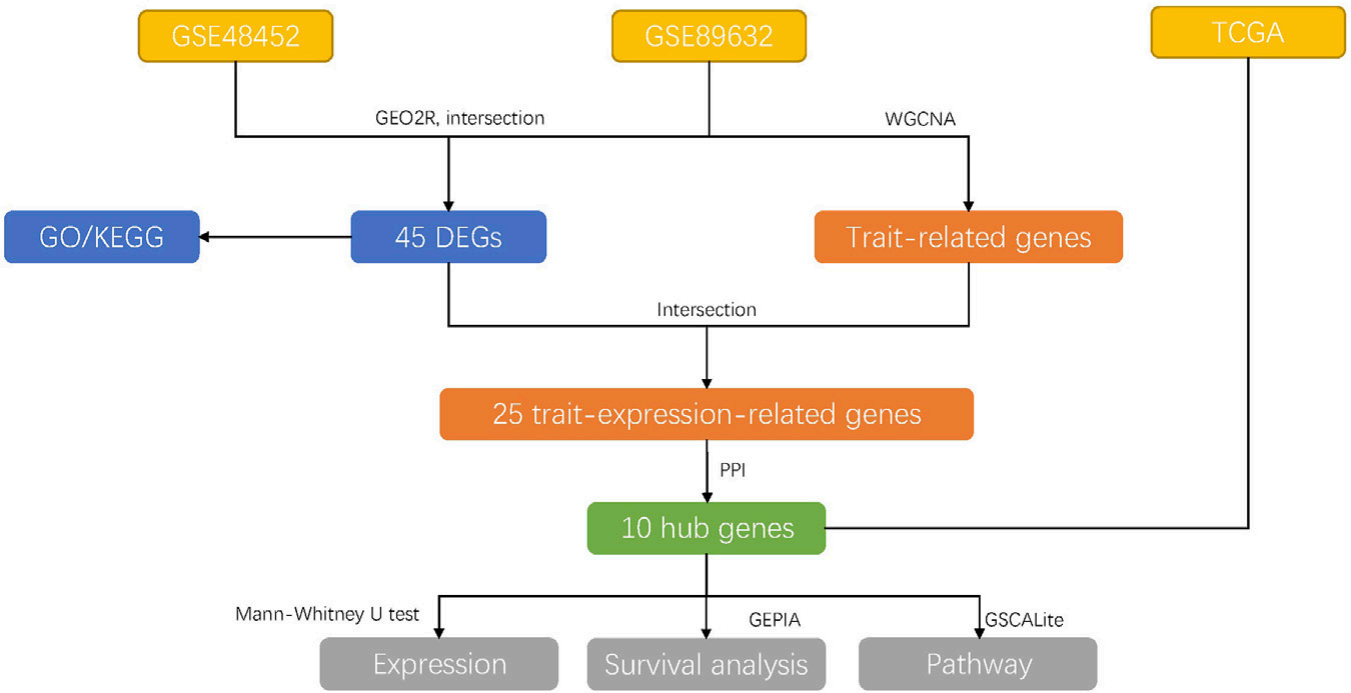
The overall research designs. The data were downloaded from GEO and TCGA databases. Then, the DEGs were explored among the groups using GEO2R and were performed GO and KEGG analysis later, respectively. Subsequently, the GSE89632 dataset was used for WGCNA analysis to explore the trait-related genes, which were intersected with the DEGs to find trait-expression-related genes. PPI network analysis was performed to detect hub genes. Then, the expression, survival rate, and pathway of 10 hub genes were explored in the TCGA database.

For the analysis of DEGs, we used the GEO2R (https://www.ncbi.nlm.nih.gov/geo/geo2r/) to generate the R script, which used two R packages (GEOquery and limma). The threshold for the DEGs was set as *p*-value <0.05 and |log_2_ fold change (FC) | ≥ 1. Heat maps were drawn using R package “pheatmap”. Venn diagram was performed using the jvenn tool (http://jvenn.toulouse.inra.fr/app/example.html), and the overlaps represented the intersection between the two datasets. Figure 1 illustrated the overall research design.

### Diagnostic Methods of Different States of NAFLD

All the samples in GSE48452 and GSE89632 were validated using histological examination by a board-certified pathologist before molecular analysis, and hematoxylin and eosin (H&E) and chromotrope aniline blue (CAB) stained sections were used for histological analysis. The different states of NAFLD were diagnosed using criteria from NAFLD Activity Score (NAS) (Kleiner et al., 2005).

### Gene Ontology Analysis and Kyoto Encyclopedia of Genes and Genomes Pathway Enrichment Analysis

GO is a commonly used bioinformatics tool that supply comprehensive information on gene function of individual genomic products based on defined features and is primarily divided into three parts, molecular function (MF), biological process (BP), and cellular component (CC). KEGG is a database resource for understanding high-level biological functions and utilities. To identify the function of DEGs, GO and KEGG analysis were performed using Metascape (metascape.org) database with default settings. We determined that results were statistically significant at a level of less than 0.05 using a *p*-value. Then, histograms and bubble plots were generated with R package “ggplot2”.

### Weighted Gene Co-Expression Network Analysis

WGCNA is a well-established method for studying biological networks and diseases (Rasmussen et al., 2020). Considering that GSE89632 had more comprehensive and complete data, we used GSE89632 to detect modules highly correlated with NAFLD, and WGCNA was performed using R package “WGCNA” and carried out on all genes. The scale-free topology of the networks was assessed for various values of the β shrinkage parameter, and we chose β = 5 based on scale-free topology criterion. Finally, the dynamic tree cut algorithm was applied to the dendrogram for module identification with the mini-size of module gene numbers set as 50, and similar modules were merged following a height cutoff of 0.05. In the module-trait analysis, gene-trait significance (GS) value >0.3 and module membership (MM) value >0.55 were defined as a threshold (Zeng et al., 2020).

### Protein-Protein Interaction Network Construction

Metascape (metascape.org) database was used to construct a protein-protein interaction (PPI) network with default settings. Disconnected nodes in the network were deleted. Then, the Cytoscape software (v3.8.2) was utilized to visualize the PPI network. We used CytoHubba plugin to identify the hub genes through molecular complex detection (MCC) (Chin et al., 2014).

### Relationship Between Hub Gens in NAFLD and Hepatocellular Carcinoma Prognosis

The pathway activity was acquired from GSCALite: A Web Server for Gene Set Cancer Analysis (http://bioinfo.life.hust.edu.cn/web/GSCALite/), the survival analysis was collected from Gene Expression Profiling Interactive Analysis (GEPIA, http://gepia.cancer-pku.cn/), and the immunohistochemical pictures were collected from the Human Protein Atlas (HPA, https://www.proteinatlas.org/) database.

### Statistical Analysis

Statistical analysis was performed using R software (Version 4.1.0). Statistical comparisons between groups of normalized data were performed using the t-test or Mann-Whitney U-test according to the test condition. A difference with *p* < 0.05 was considered significant.

## Results

### Identification of DEGs in the NAFLD Patients

**FIGURE 2:**
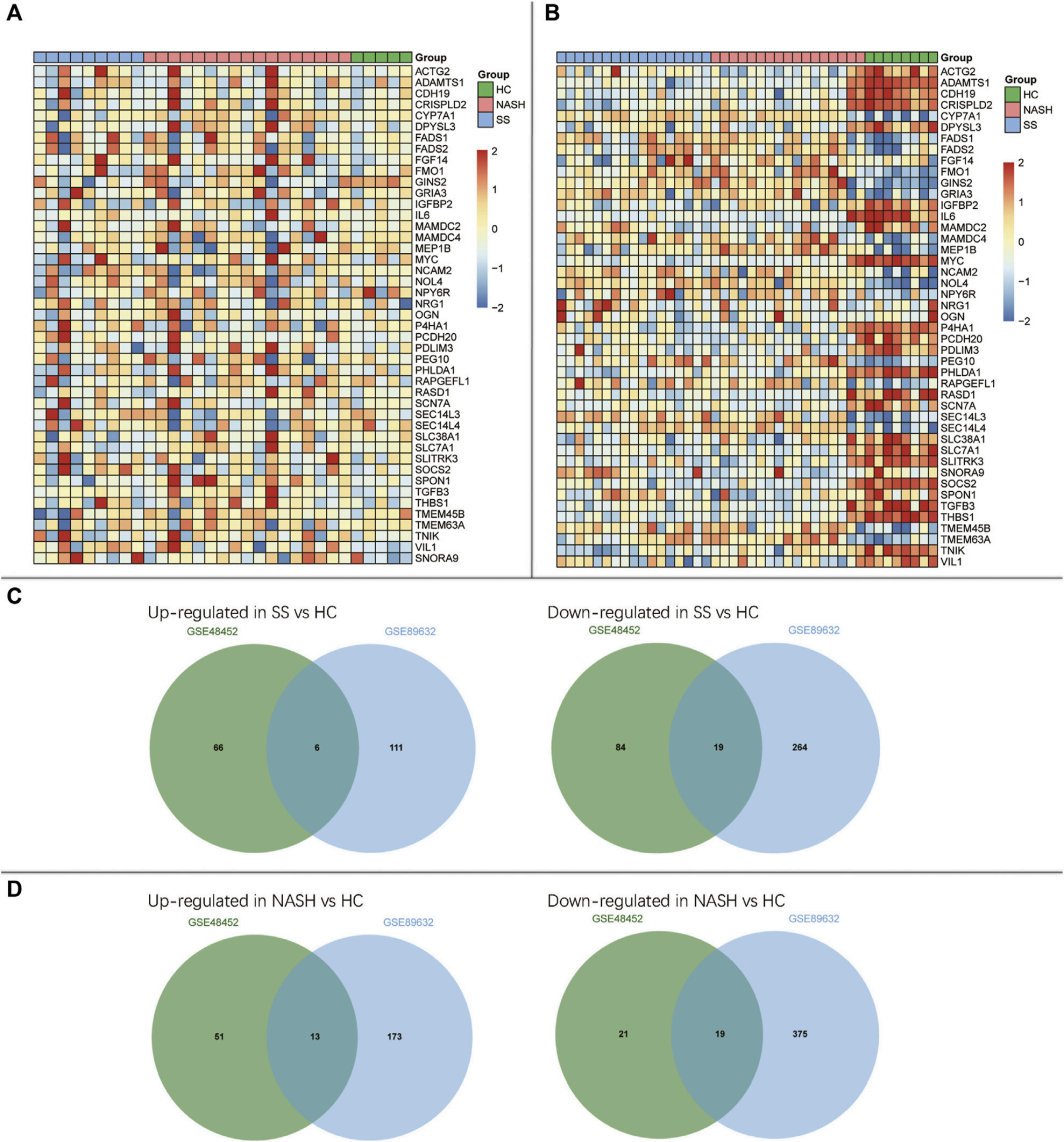
Identification of differentially expressed genes (DEGs) among HC, SS, and NASH. **(A)** Heatmap of overlapping DEGs in GSE48452; **(B)** Heatmap of overlapping DEGs in GSE89632; **(C)** Venn diagrams displayed the overlapping DEGs of up- and down-regulated genes between HC and SS; **(D)** Venn diagrams displayed the overlapping DEGs of up- and down-regulated genes between HC and NASH.

The DEGs among HC, SS, and NASH in GSE48452 and GSE89632 datasets were identified, respectively (Figures 2A,B and Supplementary Tables S1–S2). Then, we sought for the overlapping DEGs between the two datasets. We observed 6 up- and 19 down-regulated genes in SS compared with HC (Figure 2C). We also found 13 up- and 19 down-regulated genes in NASH compared with HC (Figure 2D).

### GO and KEGG Pathway Enrichment Analysis

To explore the potential roles of DEGs among HC, SS, and NASH, GO and KEGG pathway enrichment analysis were performed. The up-regulated genes between HC and SS were too few to allow identification of GO and KEGG pathway enrichment analysis, and the up-regulated genes between HC and NASH failed to enrich pathway in KEGG.

**FIGURE 3:**
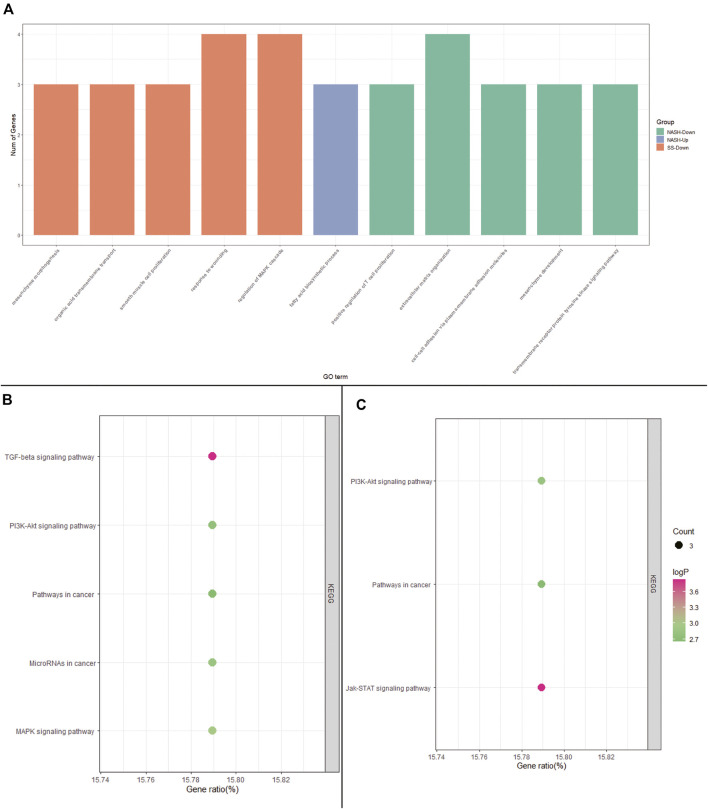
GO and KEGG pathway enrichment analysis. **(A)** GO analysis of DEGs among HC, SS, and NASH; **(B)** KEGG analysis of down-regulated DEGs between HC and SS; **(C)** KEGG analysis of down-regulated DEGs between HC and NASH.

GO analysis showed that the down-regulated genes between HC and SS were mainly involved in biological processes (BP) associated with the mesenchyme morphogenesis, organic acid transmembrane transport, smooth muscle cell proliferation, response to wounding, and regulation of MAPK cascade (Figure 3A and Supplementary Table S3). KEGG analysis indicated that the down-regulated genes between HC and SS primarily enriched in TGF-beta signaling pathway, MAPK signaling pathway, MicroRNAs in cancer, PI3K-Akt signaling pathway, and pathways in cancer (Figure 3B and Supplementary Table S4).

The DEGs between HC and NASH were mainly involved in biological processes (BP) associated with fatty acid biosynthetic process, positive regulation of T cell proliferation, extracellular matrix organization, cell-cell adhesion via plasma-membrane adhesion molecules, mesenchyme development, and transmembrane receptor protein tyrosine kinase signaling pathway (Figure 3C and Supplementary Table S5). KEGG analysis indicated that the DEGs between HC and NASH were primarily enriched in Jak-STAT signaling pathway, PI3K-Akt signaling pathway, and pathways in cancer (Figure 3D and Supplementary Table S6).

### Identification of Key Modules by WGCNA

**FIGURE 4:**
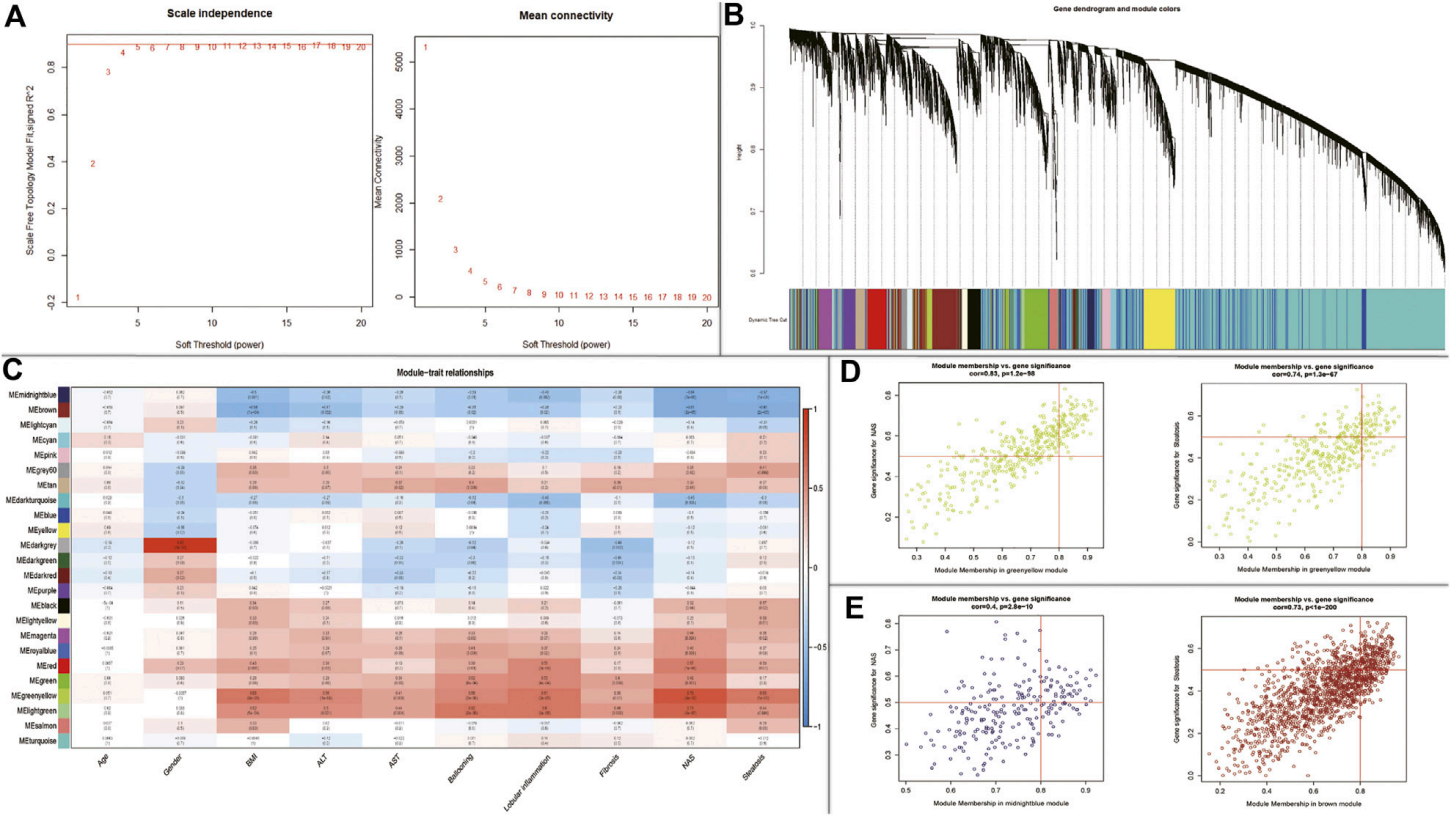
WGCNA to identify trait-related modules and genes. **(A)** Calculating soft-thresholding power; **Left:** scale-free fit indices using different soft-thresholding powers; **Right:** mean connectivity using different soft-thresholding powers; **(B)** The dendrogram clustered by Dynamic Tree Cut algorithm; **(C)** The heatmap profiling the correlations between module eigengenes and the clinical characteristics; **(D)** Scatter plot of gene significance for NAS and steatosis (Up-regulated); **(E)** Scatter plot of gene significance for NAS and steatosis (Down-regulated).

WGCNA was performed to identify key modules related to clinical traits by using GSE89632 dataset. The power of β = 5 (scale-free R^2^ = 0.89) was selected as the soft thresholding parameter to construct a scale-free network (Figure 4A). A total of 24 modules were identified (Figure 4B). Similar module clustering was constructed by using dynamic hybrid cutting (threshold = 0.05). The results in Figure 4C showed that the greenyellow module was the highest positive module correlated to NAFLD activity score (NAS, R^2^ = 0.79, *p* = 9e^−10^) and steatosis (R^2^ = 0.63, *p* = 1e^−5^). In addition, the midnightblue module was highly negative correlated to NAS (R^2^ = 0.64, *p* = 7e^−6^), and the brown module was highly negative correlated to steatosis (R^2^ = 0.61, *p* = 2e^−5^). Figures 4D,E showed the positive and negative modules.

In the module-trait analysis, we intersected the trait-related genes highly associated with NAS and steatosis and 45 DEGs generated from expression difference analysis, and finally extracted 25 trait-expression-related genes for the following analysis (Supplementary Table S7–S8).

### Identification of Hub Genes and Construction of Protein-Protein Interaction Network

**FIGURE 5:**
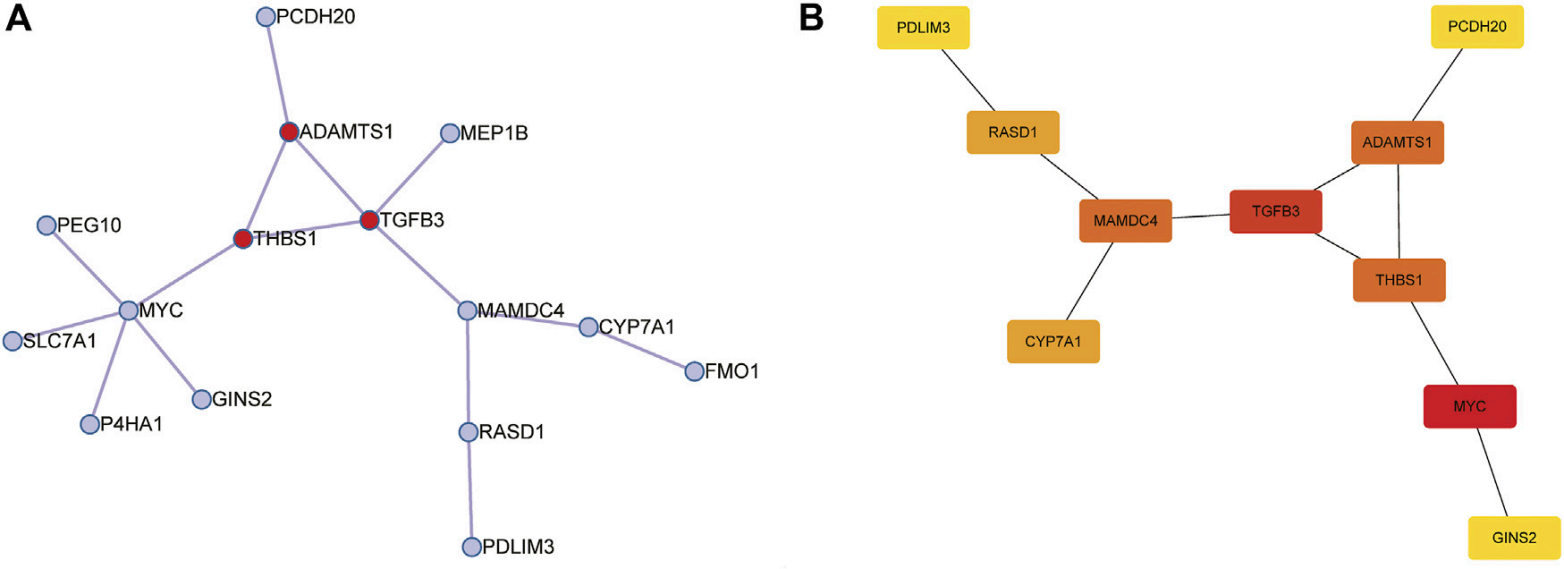
Construction of protein-protein interaction (PPI) networks for 25 trait-expression-related genes in the Metascape database. **(A)** PPI network of 15 trait-expression-related genes. Red node represented the Molecular Complex Detection (MCODE) algorithm applied to identify densely connected network components. **(B)** Results of the CytoHubba plugin; the color changed from yellow to red was indicative of the rank of protein, and the deeper the red staining, the higher rank of protein was.

Subsequently, we construct a PPI network with 25 trait-expression-related genes in the Metascape database. Then, 15 filtered genes were identified (Figure 5A) and later imported into CytoHubba plugin to explore the hub genes by “MCC” methods. The results showed that *MYC*, *TGFB3*, *ADAMTS1*, *THBS1*, *RASD1*, *PCDH20* (Down-regulated genes), *MAMDC4*, *CYP7A1*, *GINS2*, and *PDLIM3* (Up-regulated genes) were the top 10 hub genes (Figure 5B).

### Hub Genes in NAFLD Were Associated With Hepatocellular Carcinoma Prognosis

**FIGURE 6:**
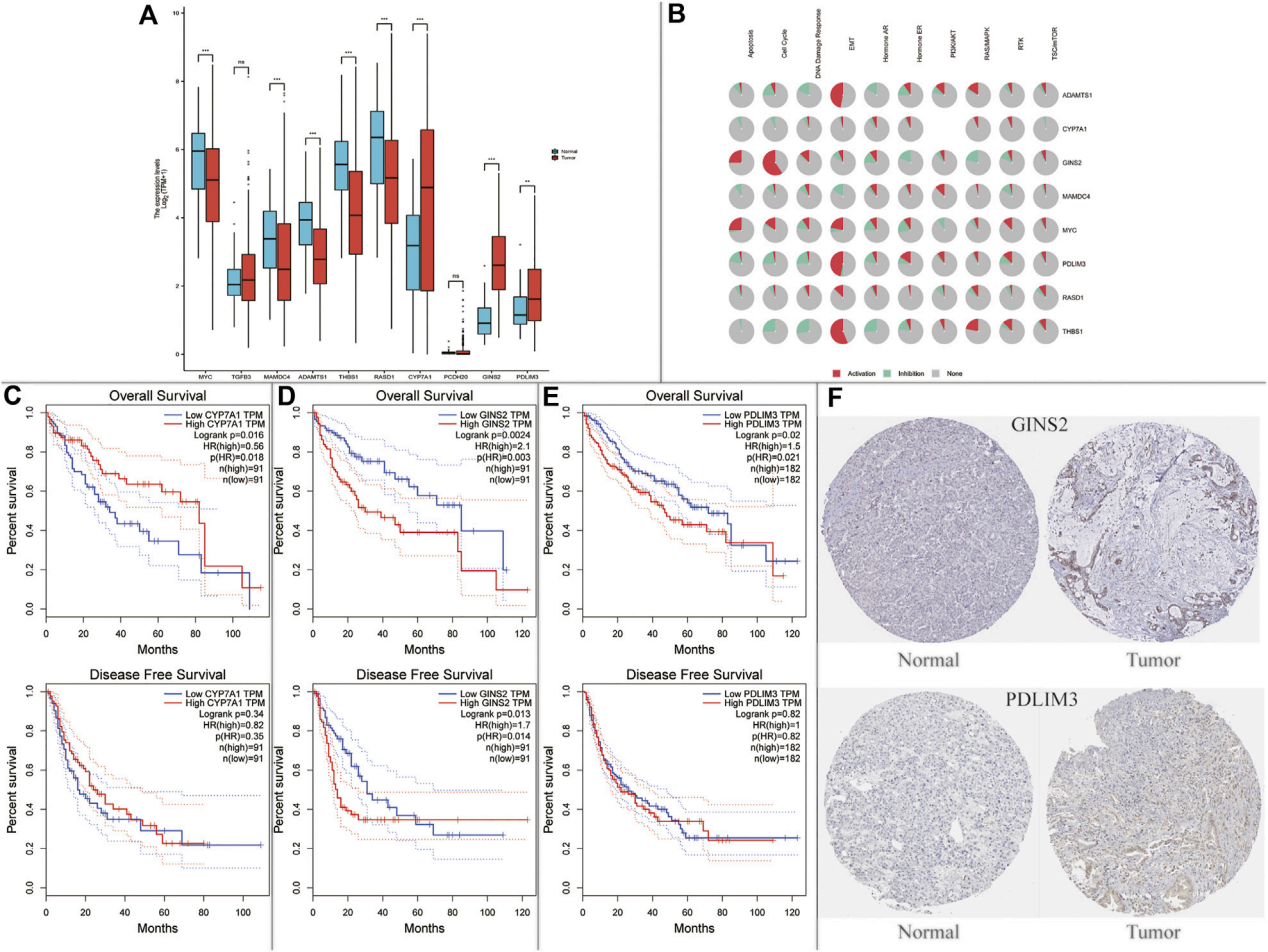
Expression and survival analysis of the NAFLD’s hub genes in hepatocellular carcinoma (HCC). **(A)** Hub genes in NAFLD were dysregulated in hepatocellular carcinoma (ns, not significant; *p < 0.05; **p < 0.01; ***p < 0.001); **(B)** Enriched pathways of 10 hub genes in the TCGA database. **(C–E)** Survival plots of *CYP7A1*, *GINS2*, and *PDLIM3*; **(F)** Protein expression of *GINS2* and *PDLIM3* between normal patients and HCC patients in the HPA database.

Afterwards, the possible relationship between hub genes and hepatocellular carcinoma (HCC) was explored. We found that *CYP7A1*, *GINS2*, and *PDLIM3* were significantly up-regulated, and *MYC*, *MAMDC4*, *ADAMTS1*, *THBS1*, and *RASD1* were significantly down-regulated in HCC tumor samples compared with normal samples using the TCGA dataset (Figure 6A). Moreover, we found that the 8 genes above were enriched in tumor-related pathways, such as apoptosis, cell cycle, and epithelial-mesenchymal transition (EMT) (Figure 6B). Subsequently, we performed survival analysis in the genes above. As demonstrated in Figure 6C, *CYP7A1*-high (using quartile cutoff points) patients showed higher overall survival (OS) rates compared to *CYP7A1*-low patients but had no effects on disease free survival rate (DFS). What is more, compared to *GINS2*- high (using quartile cutoff points) and *PDLIM3*-high (using median cutoff points) patients, the OS rates were higher in low expression patients. In addition, *GINS2*-low patients showed a higher DFS rate compared to *GINS2*-high patients (Figures 6D,E). In the HPA database, the expression of *CYP7A1*/*GINS2*/*PDLIM3* was also abnormally elevated in HCC, but the immunohistochemical picture of *CYP7A1* was missing. (Figure 6F).

## Discussion

Currently, the pathogenesis of NAFLD is still unclear, and the therapeutic treatments are also limited. In the present study, we identified 45 intersected DEGs between HC-SS group and HC-NASH group, and respectively performed GO and KEGG pathway enrichment analysis to explore the potential effects of these DEGs in NAFLD. The results showed that the GO enrichments were involved in fatty acid metabolism, mesenchyme, extracellular matrix, cell adhesion, and inflammatory and immune response, which also played important roles in tumorigenesis. KEGG analysis showed that the DEGs were primarily enriched in TGF-beta signaling pathway, PI3K-Akt signaling pathway, pathways in cancer, MicroRNAs in cancer, MAPK signaling pathway, and Jak-STAT signaling pathway. Both the results of GO and KEGG analysis all pointed to tumorigenesis. Meanwhile, the overlapping pathways between SS and NASH were PI3K-Akt signaling pathway and pathways in cancer, suggesting that the two pathways could be an important therapeutic target for NAFLD. The PI3K-AKT signaling pathway is known for regulating metabolism, cell growth, and cell survival. The active form of PI3K is an oncogene; thus, amplification and mutations of PI3K are usually found in many kinds of cancers (Matsuda et al., 2013). However, in this study, the PI3K-AKT signaling pathway was down-regulated in NAFLD patients. Previous studies had shown that the inhibition of PI3K-AKT signaling pathway increased hepatic insulin resistance, which exacerbated the accumulation of fat in the liver (Ntandja et al., 2020); what’s more, a restoration of PI3K-AKT pathway improved the liver injury and fat accumulation (Li et al., 2013). Long-duration effects of lipotoxicity aggravated the inflammatory reaction in the liver, leading to dysregulation of the PI3K-AKT signaling pathway, which might finally result in HCC (Asgharpour et al., 2016). Our findings were also consistent with previous reports (Wang et al., 2019; Liu et al., 2020).

Due to NAS and steatosis were the two main pathologic indicators in the estimation of NAFLD, we tried to find out the DEGs related to the NAS and steatosis. We identified 25 DEGs related to the NAS and steatosis, and PPI network analysis was performed to explore the hub genes in the pathogenesis and progression of NAFLD. Eventually, we determined 10 hub genes (Down-regulated genes: *MYC*, *TGFB3*, *ADAMTS1*, *THBS1*, *RASD1*, *PCDH20*; Up-regulated genes: *MAMDC4*, *CYP7A1*, *GINS2*, and *PDLIM3*) related to NAS and steatosis.

HCC is the fourth-leading cause of cancer death worldwide, and the morbidity of NAFLD-related HCC is predicted to increase dramatically by 2030, with increases of 82, 117, and 122% from 2016 in China, France, and the USA, respectively (Yang et al., 2019; Huang et al., 2021). Therefore, we explore whether these ten hub genes were associated with the progression in HCC in the TCGA database. We found that *CYP7A1*, *GINS2*, and *PDLIM3* were significantly up-regulated, and *MYC*, *MAMDC4, ADAMTS1*, *THBS1*, and *RASD1* were significantly down-regulated in HCC tumor samples compared to normal samples. Surprisingly, we also found that *CYP7A1*/*GINS2*/*PDLIM3* were correlated with HCC prognosis.

*CYP7A1*, catalyzing the first and rate-limiting step in the classic bile acid synthesis pathway, has been shown to be involved in lipid metabolism (Wang et al., 2020). Deficiency of *CYP7A1* caused by homozygous deletion mutations can inhibit the production of bile acids, leading to the accumulation of cholesterol in the liver, reducing LDL receptors and elevating LDL cholesterol (Pullinger et al., 2002). However, *CYP7A1* was up-regulated in SS and NASH group compared with HC group in our study. Previous studies have shown that *CYP7A1* and its associated cholesterol processes were adversely regulated in NAFLD (Wruck and Adjaye, 2017), and glucose stimulates *CYP7A1* transcription in human hepatocytes (Chiang and Ferrell, 2020). Therefore, up-regulating CYP7A1 in NAFLD may be the consequence rather than the cause of disease (Jia and Zhai, 2019). In addition, increased *CYP7A1* expression and bile acid synthesis ameliorated hepatic inflammation and fibrosis, proving its anti-tumor effects (Liu et al., 2016).

*GINS2*, a member of the *GINS* family, plays a crucial role in DNA duplication and is highly expressed in various types of cancer (Kubota et al., 2003; Tian et al., 2020). However, very little research can be found about *GINS2* in the liver, especially in NAFLD. Previous bioinformatics studies indicated that *GINS2* might be the hub genes in the development of NASH to HCC and predicted poor prognosis in HCC, but there was no further experiment to verify its effects on NAFLD (Lian et al., 2018; Zhang et al., 2020).

*PDLIM3*, highly expressed in skeletal and cardiac muscle, has been suggested to play a pivotal role in myocyte stability, signal transduction, and mechanical signaling, especially in growth and remodeling processes (Zheng et al., 2010). Interestingly, *PDLIM3* was firstly screened out for a new hub gene in the pathogenesis of NAFLD and was associated with the prognosis of HCC. *PDLIM3* was highly related to EMT in the GSCALite database, which might partially reveal its effects in the pathogenesis in NAFLD and HCC. More future studies are needed to gain more insights about *PDLIM3*.

In the present study, more attention was applied to the pathogenesis of NAFLD in obesity, which was rare in other studies. However, the present study had several limitations. Firstly, further experiments were required to verify these results. Secondly, it was hard to identify HCC patients caused by NAFLD in the TCGA database, which might impact the outcomes.

In conclusion, we analyzed two public datasets to identify DEGs among HC, SS and NASH. GO and KEGG pathway analysis revealed that the pathogenesis and progression of NAFLD were highly associated with tumorigenesis. Finally, we screened out 10 hub genes related to NAS and steatosis, and three of them were correlated with HCC prognosis. Although further validation is still needed, we provide useful and novel information to explore the potential candidate genes for NAFLD prognosis and therapeutic options.

## Data Availability

The datasets presented in this study can be found in online repositories. The names of the repository/repositories and accession number(s) can be found in the article/Supplementary Material.
