# Supplementary material for: Bioinformatics Analysis Explores Potential Hub Genes in Nonalcoholic Fatty Liver Disease
Source: Front Genet. 2021 Oct 29;12:772487. doi: 10.3389/fgene.2021.772487 (PMC8586215; doi:10.3389/fgene.2021.772487)
Supplement: Supplementary file 3 [file Table5.DOCX]

**Table S5** GO analysis of DEGs between HC and NASH

| **Category** | **Description** | **LogP** | **Enrichment** | **Z-score** | **Count** | **GeneRatio** | **Hits** | **P value** |
| --- | --- | --- | --- | --- | --- | --- | --- | --- |
| **Up-regulated** |  |  |  |  |  |  |  |  |
| Biological Processes | fatty acid biosynthetic process | -4.28248 | 39.86786 | 10.6953 | 3 | 23.07692 | CYP7A1\|FADS1\|FADS2 | 5.22E-05 |
| Molecular Functions  **Down-regulated** | oxidoreductase activity | -3.54411 | 11.64599 | 6.324623 | 4 | 30.76923 | CYP7A1\|FMO1\|FADS1\|FADS2 | 2.86E-04 |
| Biological Processes | positive regulation of T cell proliferation | -4.38071 | 44.02293 | 11.25486 | 3 | 15.78947 | IGFBP2\|IL6\|SLC7A1 | 4.16E-05 |
| Biological Processes | extracellular matrix organization | -4.35395 | 19.63053 | 8.458034 | 4 | 21.05263 | IL6\|P4HA1\|ADAMTS1\|CRISPLD2 | 4.43E-05 |
| Biological Processes | cell-cell adhesion via plasma-membrane adhesion molecules | -3.09963 | 16.16842 | 6.568059 | 3 | 15.78947 | SLITRK3\|CDH19\|PCDH20 | 7.95E-04 |
| Biological Processes | mesenchyme development | -3.02435 | 15.22711 | 6.349958 | 3 | 15.78947 | ACTG2\|IL6\|MYC | 9.45E-04 |
| Biological Processes | transmembrane receptor protein tyrosine kinase signaling pathway | -2.0845 | 7.068865 | 3.999792 | 3 | 15.78947 | IGFBP2\|VIL1\|SOCS2 | 8.23E-03 |
| Cellular Components | actin cytoskeleton | -2.34097 | 8.75259 | 4.581747 | 3 | 15.78947 | ACTG2\|VIL1\|PDLIM3 | 4.56E-03 |
