# Supplementary material for: Bioinformatics Analysis Explores Potential Hub Genes in Nonalcoholic Fatty Liver Disease
Source: Front Genet. 2021 Oct 29;12:772487. doi: 10.3389/fgene.2021.772487 (PMC8586215; doi:10.3389/fgene.2021.772487)
Supplement: Supplementary file 4 [file Table3.DOCX]

**Table S3** GO analysis of down-regulated genes between HC and SS

| **Category** | **Description** | **LogP** | **Enrichment** | **Z-score** | **Count** | **GeneRatio** | **Hits** | **P value** |
| --- | --- | --- | --- | --- | --- | --- | --- | --- |
| Biological Processes | mesenchyme morphogenesis | -5.17512 | 80.84211 | 15.40058 | 3 | 15.78947 | ACTG2\|MYC\|TGFB3 | 6.68E-06 |
| Biological Processes | organic acid transmembrane transport | -3.8702 | 29.64211 | 9.139231 | 3 | 15.78947 | SLC7A1\|THBS1\|SLC38A1 | 1.35E-04 |
| Biological Processes | smooth muscle cell proliferation | -3.60869 | 24.16476 | 8.191427 | 3 | 15.78947 | OGN\|TGFB3\|THBS1 | 2.46E-04 |
| Biological Processes | response to wounding | -3.3488 | 10.73989 | 6.005123 | 4 | 21.05263 | DPYSL3\|NRG1\|TGFB3\|THBS1 | 4.48E-04 |
| Biological Processes | regulation of MAPK cascade | -3.02379 | 8.795877 | 5.32298 | 4 | 21.05263 | NRG1\|MYC\|THBS1\|TNIK | 9.47E-04 |
| Molecular Functions | growth factor activity | -5.41747 | 36.59519 | 11.80595 | 4 | 21.05263 | FGF14\|NRG1\|OGN\|TGFB3 | 3.82E-06 |
| Molecular Functions | extracellular matrix structural constituent | -3.69484 | 25.85067 | 8.494388 | 3 | 15.78947 | OGN\|THBS1\|SPON1 | 2.02E-04 |
| Molecular Functions | glycosaminoglycan binding | -3.31409 | 19.16515 | 7.219029 | 3 | 15.78947 | DPYSL3\|THBS1\|CRISPLD2 | 4.85E-04 |
| Molecular Functions | growth factor binding | -3.94062 | 31.31208 | 9.409294 | 3 | 15.78947 | IGFBP2\|TGFB3\|THBS1 | 1.15E-04 |
| Molecular Functions | cell adhesion molecule binding | -2.25826 | 8.173375 | 4.389936 | 3 | 15.78947 | NRG1\|THBS1\|CDH19 | 5.52E-03 |
| Cellular Components | extracellular matrix | -4.513 | 13.00092 | 7.521282 | 5 | 26.31579 | OGN\|TGFB3\|THBS1\|SPON1\|CRISPLD2 | 3.07E-05 |
| Cellular Components | secretory granule lumen | -2.90207 | 13.80843 | 6.006488 | 3 | 15.78947 | TGFB3\|THBS1\|CRISPLD2 | 1.25E-03 |
| Cellular Components | apical plasma membrane | -4.01675 | 16.06618 | 7.569722 | 4 | 21.05263 | IGFBP2\|SLC7A1\|TNIK\|SLC38A1 | 9.62E-05 |
| Cellular Components | cell body | -3.27347 | 10.25678 | 5.842878 | 4 | 21.05263 | ACTG2\|DPYSL3\|TGFB3\|SLC38A1 | 5.33E-04 |
| Cellular Components | axon | -2.04371 | 6.829978 | 3.91053 | 3 | 15.78947 | DPYSL3\|NRG1\|SLC38A1 | 9.04E-03 |
