# Supplementary material for: Bioinformatics Analysis Explores Potential Hub Genes in Nonalcoholic Fatty Liver Disease
Source: Front Genet. 2021 Oct 29;12:772487. doi: 10.3389/fgene.2021.772487 (PMC8586215; doi:10.3389/fgene.2021.772487)
Supplement: Supplementary file 7 [file Table4.DOCX]

**Table S4** KEGG analysis of down-regulated genes between HC and SS

| **Category** | **Description** | **LogP** | **Enrichment** | **Z-score** | **Count** | **GeneRatio** | **Hits** | **P value** |
| --- | --- | --- | --- | --- | --- | --- | --- | --- |
| KEGG | TGF-beta signaling pathway | -4.62035 | 52.93233 | 12.38584 | 3 | 15.78947 | MYC\|TGFB3\|THBS1 | 2.40E-05 |
| KEGG | MAPK signaling pathway | -3.19468 | 17.43653 | 6.851015 | 3 | 15.78947 | FGF14\|MYC\|TGFB3 | 6.39E-04 |
| KEGG | MicroRNAs in cancer | -2.99468 | 14.87062 | 6.2654 | 3 | 15.78947 | MYC\|SLC7A1\|THBS1 | 1.01E-03 |
| KEGG | PI3K-Akt signaling pathway | -2.82702 | 13.00092 | 5.802036 | 3 | 15.78947 | FGF14\|MYC\|THBS1 | 1.49E-03 |
| KEGG | Pathways in cancer | -2.6486 | 11.2565 | 5.334139 | 3 | 15.78947 | FGF14\|MYC\|TGFB3 | 2.25E-03 |
