# Supplementary material for: Bioinformatics Analysis Explores Potential Hub Genes in Nonalcoholic Fatty Liver Disease
Source: Front Genet. 2021 Oct 29;12:772487. doi: 10.3389/fgene.2021.772487 (PMC8586215; doi:10.3389/fgene.2021.772487)
Supplement: Supplementary file 8 [file Table6.DOCX]

**Table S6** KEGG analysis of down-regulated genes between HC and NASH

| **Category** | **Description** | **LogP** | **Enrichment** | **Z-score** | **Count** | **GeneRatio** | **Hits** | **P value** |
| --- | --- | --- | --- | --- | --- | --- | --- | --- |
| KEGG | Jak-STAT signaling pathway | -3.81987 | 28.50202 | 8.950197 | 3 | 15.78947 | IL6\|MYC\|SOCS2 | 1.51E-04 |
| KEGG | PI3K-Akt signaling pathway | -2.82702 | 13.00092 | 5.802036 | 3 | 15.78947 | FGF14\|IL6\|MYC | 1.49E-03 |
| KEGG | Pathways in cancer | -2.6486 | 11.2565 | 5.334139 | 3 | 15.78947 | FGF14\|IL6\|MYC | 2.25E-03 |
